# Supplementary material for: Do buyers have bargaining power? Evidence from informal groundwater contracts
Source: PLoS One. 2020 Sep 30;15(9):e0236696. doi: 10.1371/journal.pone.0236696 (PMC7527252; doi:10.1371/journal.pone.0236696)
Supplement: S1 File — (DOCX) [file pone.0236696.s001.docx]

**Supplementary material**

1. **Experimental Instructions**

Enumerator name: ………………………………….. Date:………….

*[This is the instruction-cum-questionnaire for the enumerator to read in front of subjects and explain in case if they do not understand. The instruction in the parenthesis is for the enumerator's actions.]*

**Message to subject**

Hi, my name is ………………………, we are from the Indian Statistical Institute (ISI), New Delhi. I hope you remember me or my colleague who conducted an interview with you in the month of April-May this year. We would like to conduct another interview with you. You will be faced with some decision-making situations with other members of the village. All the decisions involve real money. If you decide to participate we will pay you Rs. 100 in order to compensate you for your time. Furthermore, you might earn additional money by participating. How much depends on your and other participant decisions.

The interview runs in two phases. In the first phase, you will make some decisions here and now. In the second phase, you will make some more decisions here and as a part of the second phase, you also have to come to ……………. Place at ……… an hour to make additional decisions, some of them together with another member from this village. Soon after the second phase, you will be paid the participation fee as well as the additional amount of money you might have earned by participating and making decisions. In total, it takes about one and a half hours to complete the interview.

Whatever the information you provide it will be kept anonymous and only reported in an aggregated form where no individuals can be identified.

Would you like to participate now?

*[If now continue with the following sections]*

*[If not now, ask the available time and come back at the stated time]*

Are you a seller or a buyer in the previous water contract? Seller Buyer

Your name ………………………….. S/0 ………………………………

Participant ID……………………….. *[From the previous survey list]*

Village: ………………………………

**Phase I**

1. **Introduction**

From our previous survey on groundwater market we observed mainly two types of water contracts in this area:

1. Output-Shared Contract and,
2. Fixed Contract

You are currently a seller of water contracts. You might have only one of these contracts, or both types, or some other type. We are in this survey interested to know what contract you would like to have under different circumstances. You will be asked to choose what contract you would like to have given these circumstances. The situations that are presented might differ from your present conditions. They may not apply to you in terms of the crop grown, or area cultivated, or contract you have chosen, or contract characteristics specified, and you may not have faced such a low or high price at any time. But we would like you to imagine yourself in the given situation and to choose the contract you prefer.

You will face with two series of decisions regarding the contract choice in this phase. One series of the decision will be taken now and the other series of the decision will be taken when you come to ……… place at …..… hour. A decision from either one of the series of decisions will be randomly selected for actual payment. Like you …………. number of sellers are also approached to take decisions in this region. Out of the…… number of sellers, only one seller will be selected for real payment of a decision. However, for now, we don’t know which of the decision will be paid for real and who the seller is. Once we finish interviewing all the sellers in this region, we randomly select a seller and approached to payout a random decision. If you get selected, you will be approached after………[date] to receive your money. Therefore, you need to take decisions seriously.

I will now describe the first setting under which you will make your choices.

1. **Decision situation**

Think of a normal production year with no extreme weather events, no pest, and no disease instance. Given the land productivity and production practices in the region, on average in 10 Guntas of mulberry can yield about 50 kg of cocoons per crop season.

Think of a situation where you as a seller is being approached by a buyer who has 10 Guntas of land and plans to grow the Mulberry crop for the coming season. The buyer wishes to have a water contract with you. For a shared contract the buyer agrees to pay a water price of one-third of the total revenue. For a fixed contract the buyers agree to pay Rs. 4000 per season. In both cases, you have to provide water to that per 10 guntas during one season.

Now, imagine a situation where the marketing board is working to help farmers by providing them information about the expected future crop prices. This information will be provided to the village before the season starts. The prices are, however, not known for sure. Instead, the marketing board provides a high and a low price, and their best guess of what the chances are for each of these two prices. For example, it will be represented as; with 30% chance price will be lowest and with a 70% chance price will be highest in the market.

Let me explain this with an example *[Show the figure below].* This box consists of ten slips. Out of ten, 3 of them are red representing a low price and 7 of them are green representing a high price. If one randomly picks a slip from the box, there is a 30% chance that it is a red slip and a 70% chance that it is a green slip. That is, at least there will be one red slip if you pick three slips randomly.


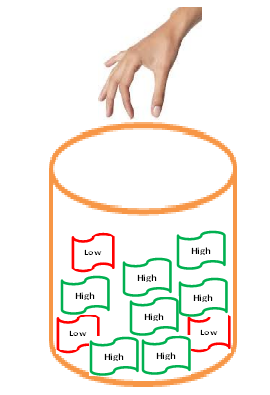


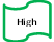
high price


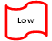
Low price

Do you have any questions?

1. **Test questions:**

Now I will begin by asking you some questions about this before we continue.

If the marketing board announces “With 10% chance the price will be low, and with 90% chance the price will be high per kg of cocoons”

In this case how many of the ten slips do you think are green? Remember that green is the high price. *[Circle the respondent answer]*

Ans: 1, 2, 3, 4, 5, 6, 7, 8, 9, 10

In this case, how many of the ten slips do you think are red. Remember that red is the low price. *[Circle the respondent answer]*

Ans: 1, 2, 3, 4, 5, 6, 7, 8, 9, 10

*[Those who marked 9 and 1 in question 1 and 2 respectively can proceed to the next part of the questionnaire]*

*[Those who give other responses than 9 and 1, the enumerator should explain the chance of occurrence of predicted prices in the market once again and confirm their understanding from the example]*

*[Enumerator has to fill the following blank based on the above answers of the subject.*

1. *As the subject answered for the first explanations? Yes / No*
2. *If no, number explanations he took to answer correctly ……………………..*
3. *What is your belief about the subject in understanding this price explanation?*
4. *Very quick*
5. *Quick*
6. *Moderate*
7. *Slow*
8. *Very slow]*

For the coming season, the marketing board has predicted **the low price per kg of mulberry cocoons to be Rs. 100, while the high price is predicted to be Rs. 400**. Thus, the market price will either be Rs 100 or Rs 400. We will now ask you to make a choice between a shared and fixed contact. We will vary the chance of low and high prices. As I have explained the chances are for example expressed like this: “With a 30% chance, the predicted price will be Rs. 100, while with a 70% chance the predicted price will be Rs. 400 per kg of cocoons”.

Now you will be given a decision sheet, where you need to make a decision about which contract you prefer? *[Hand over the decision sheet, and explain the following text]*

Here is the decision sheet, which consists of 11 rows. Each row shows the revenue that you can obtain in a shared and fixed contract under different chances of low and high prices in the market.

For the shared contract your revenue depends on whether the market price is low or high. For an average yield of 50 kg of the cocoon from 10 guntas, the total revenue will be either Rs 20000 or Rs 5000. You will be paid 1/3^rd^ share of the total revenue for the water, and your earnings as a seller will be either Rs 6667 or Rs. 1667. For the fixed contract, your revenue is always Rs. 4000, irrespective of the market price. Therefore, you earn Rs. 4000 for sure.

In the first row, the market price will be high for sure, i.e Rs. 400, and in the second row the chance of a high market price is 90%. As you go down the rows, the chance of the high price goes down, and it will be zero at the bottom of the sheet. For the fixed contract, your earnings from selling water will be constant irrespective of the market price.

Therefore, in the first row, you for sure earn Rs. 4000 with a fixed contract, and Rs. 6667 in the shared contract. If you prefer a fixed contract we will tick this box [Show box on decision sheet], if you prefer a shared contract we will tick this box [Show box on decision sheet]. If you choose a fixed contract for the first row, you will always like a fixed contract for the other rows as well since the chance of getting a high market price for the crop goes down for the following rows. If you choose a shared contract we will then go to the next row where the chance of getting a higher market price goes down to 90%. We will then ask you to choose either a shared or fixed contract. And then continue to the next row.

Once you have switched to the shared contract, you will always prefer that for the following rows.

*[Show the following pictures to explain to the subject about how they should mark]*

Figure 1, is one way of choosing, that is you can switch only once, while figure 2, is wrong as you cannot do multiple switches from one contract to other. However, it is your choice at what point you would like to switch.


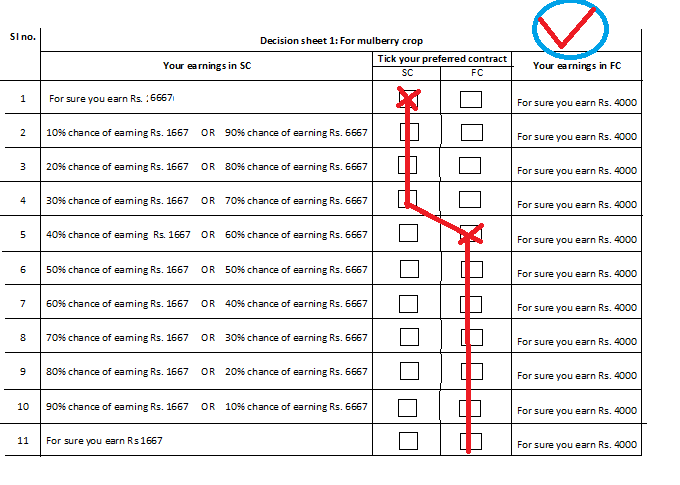


**Figure S1:** Right way of choosing


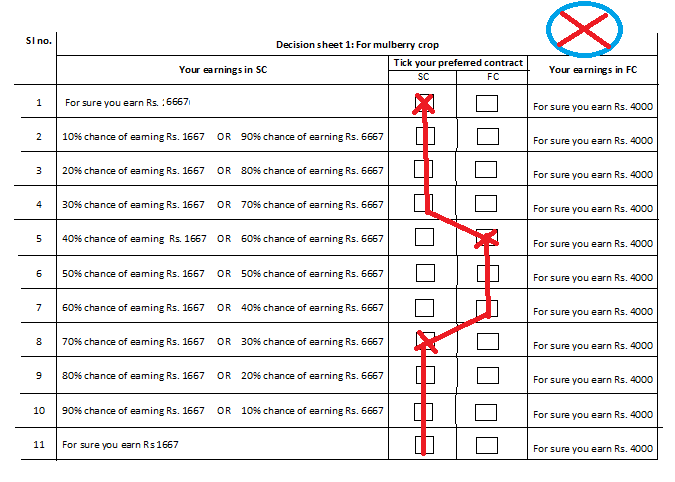


**Figure S2:** Wrong-way of choosing

If this series of decisions got selected for payment, in order to choose a decision from the set of decisions, you will be asked to choose a card from 11 decks of cards each consists of a number between 1 and 11. The numbers in the card represent the rows in the decision sheet. If you have chosen fixed contract for the selected row, then you will be paid Rs. 4000, and if you have chosen shared contract, you will be asked to pick a slip from the box consists of mixture of red and green slips which represent the corresponding chance of high and low price occurrence of the row. If it is a red slip that represented low price earning, you will be paid Rs. 1667 and if it is a green slip that represented high price earning, you will be paid Rs. 6667.

Do you have any questions?

There is no right or wrong choice here. We want to learn about when you would prefer a Fixed Contract compare to a Shared Contract.

Seller Id …………………………….. *[From the previous list]*

| **Table S1: Seller individual decision for mulberry crop** | | | | | | |
| --- | --- | --- | --- | --- | --- | --- |
| **Sl no.** | **Your earnings in SC** | | | **Tick your preferred contract** | | **Your earnings in FC** |
|  |  |  |  | SC | FC |  |
| 1 | For sure you earn Rs. 6667 | | | 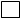 | 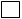 | For sure you earn Rs. 4000 |
| 2 | 10% chance of earning Rs. 1667 | OR | 90% chance of earning Rs. 6667 | 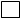 | 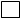 | For sure you earn Rs. 4000 |
| 3 | 20% chance of earning Rs. 1667 | OR | 80% chance of earning Rs. 6667 | 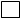 | 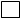 | For sure you earn Rs. 4000 |
| 4 | 30% chance of earning Rs. 1667 | OR | 70% chance of earning Rs. 6667 | 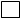 | 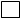 | For sure you earn Rs. 4000 |
| 5 | 40% chance of earning Rs. 1667 | OR | 60% chance of earning Rs. 6667 | 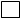 | 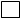 | For sure you earn Rs. 4000 |
| 6 | 50% chance of earning Rs. 1667 | OR | 50% chance of earning Rs. 6667 | 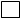 | 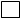 | For sure you earn Rs. 4000 |
| 7 | 60% chance of earning Rs. 1667 | OR | 40% chance of earning Rs. 6667 | 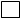 | 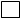 | For sure you earn Rs. 4000 |
| 8 | 70% chance of earning Rs. 1667 | OR | 30% chance of earning Rs. 6667 | 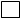 | 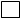 | For sure you earn Rs. 4000 |
| 9 | 80% chance of earning Rs. 1667 | OR | 20% chance of earning Rs. 6667 | 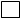 | 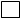 | For sure you earn Rs. 4000 |
| 10 | 90% chance of earning Rs. 1667 | OR | 10% chance of earning Rs. 6667 | 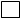 | 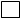 | For sure you earn Rs. 4000 |
| 11 | For sure you earn Rs 1667 | | | 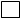 | 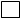 | For sure you earn Rs. 4000 |

You have now finished the first series of decisions in the second phase of the interview. As explained before, as a part of the second phase, you are asked to come to ……………….. place at ……………..time in order to make some more decisions and to receive payment.

Thank you very much for your kind cooperation. We also kindly ask you, please do not discuss with others regarding what you earn from this survey.

*[Check whether you have attached the household list and decision sheet 1 to the booklet]*

**Phase II**

Welcome again for the second phase of the interview.

In this part, we ask you to make similar decisions as before and choose between shared and fixed contracts under different chances of a low and high price. But this time, we want you to make a decision together with your buyer if you are a seller or with your seller if you are a buyer.

Therefore, we ask you to sit next to your partner in the water contract and discuss the contract scenario we presented earlier. *[If the seller has more than one buyer, start with the buyer 1, then buyer 2 buyers 3 and buyer 4 as specified in the buyer-seller matched list]*

*[Hand over the respective individual decisions sheet and give them some time to discuss]*

Like you, your contract partner was also given the same information and made his choices. You might encounter that the choices you made are different from the choices your partner/s has made. However, you both have to agree upon either one of the contracts for all the different chances of low and high prices. If the matched pair of sellers and buyers failed to agree upon the contract, the joint series of the decision will not be considered for actual payment if the buyer or seller gets selected for the payment.

Now you will be given decision sheet 2. You both have to discuss and agree upon a contract for each chance occurrence of a low and a high price.

*[Hand over the decision sheet 2]*

Decision sheet 2 is similar to the one you saw before, but now both buyer and seller earnings are presented. You can see that the shared contract earnings of the seller and the buyer depend on the market price. For a fixed contract, the earning of the buyer depends on the marker price and the earning of the seller is constant irrespective of the market price.

For an average yield of 50 kg of the cocoon from 10 guntas, the total revenue will be either Rs 20000 or Rs. 5000. One-third of the total revenue has to be paid as water price in the shared contract, therefore, the seller earns either Rs. 6667 or 1667 and the buyer earns either Rs. 13333 or Rs. 3333. In a fixed contract, Rs. 4000 has to be paid for water, therefore, the seller earns Rs. 4000 and buyer earns either Rs. 1000 or Rs. 16000.

In the first row, the market price will be high for sure i.e Rs. 400, and in the second row the chance of predicted price to be high is 90%. As you go down the rows, the chance of high price goes down, and it will be zero at the bottom of the sheet.

Therefore, in the first row for sure seller earns Rs. 6667 and the buyer earn Rs. 13333 with the shared contract, and Rs. 4000 and Rs. 16000 with the fixed contract. If you agree on a fixed contract we will tick this box [Show box on decision sheet], if you agree on the shared contract we will tick this box [Show box on decision sheet]. Then I proceed to the next row.

| **Table S2: Joint decision sheet for mulberry crop.** **Seller ID ……………………………. , Buyer ID …………………………………** | | | | | | |
| --- | --- | --- | --- | --- | --- | --- |
| **Decision row** | **Earnings in SC** | | | **Your choice** | | **Earnings in FC** |
|  |  |  |  | **SC** | **FC** |  |
| 1 | Certainty that seller earns INR **6667** and buyer earns INR **13333** | | | 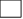 | 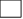 | Certainty that seller earns INR **4000** and buyer earns INR **16000** |
| 2 | With 10% chance seller earns INR **1667** and buyer earns INR **3333** | OR | With 90% chance seller earns INR **6667** and buyer earns INR **13333** | 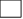 | 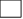 | Seller earns INR **4000** and buyer earns INR **1000** with 10% chance or INR **16000** with 90% chance |
| 3 | With 20% chance seller earns INR **1667** and buyer earns INR **3333** | OR | With 80% chance seller earns INR **6667** and buyer earns INR **13333** | 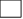 | 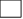 | Seller earns INR **4000** and  buyer earns INR **1000** with 20% chance or INR **16000** with 80% chance |
| 4 | With 30% chance seller earns INR **1667** and buyer earns INR **3333** | OR | With 70% chance seller earns INR **6667** and buyer earns INR **13333** | 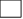 | 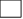 | Seller earns INR **4000** and  buyer earns INR **1000** with 30% chance or INR **16000** with 70% chance |
| 5 | With 40% chance seller earns INR **1667** and buyer earns INR **3333** | OR | With 60% chance seller earns INR **6667** and buyer earns INR **13333** | 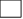 | 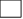 | Seller earns INR **4000** and  buyer earns INR **1000** with 40% chance or INR **16000** with 60% chance |
| 6 | With 50% chance seller earns INR **1667** and buyer earns INR **3333** | OR | With 50% chance seller earns INR **6667** and buyer earns INR **13333** | 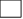 | 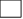 | Seller earns INR **4000** and  buyer earns INR **1000** with 50% chance or INR **16000** with 50% chance |
| 7 | With 60% chance seller earns INR **1667** and buyer earns INR **3333** | OR | With 40% chance seller earns INR **6667** and buyer earns INR **13333** | 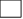 | 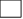 | Seller earns INR **4000** and  buyer earns INR **1000** with 60% chance or INR **16000** with 40% chance |
| 8 | With 70% chance seller earns INR **1667** and buyer earns INR **3333** | OR | With 30% chance seller earns INR **6667** and buyer earns INR **13333** | 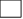 | 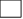 | Seller earns INR **4000** and  buyer earns INR **1000** with 70% chance or INR **16000** with 30% chance |
| 9 | With 80% chance seller earns INR **1667** and buyer earns INR **3333** | OR | With 20% chance seller earns INR **6667** and buyer earns INR **13333** | 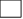 | 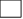 | Seller earns INR **4000** and  buyer earns INR **1000** with 80% chance or INR **16000** with 20% chance |
| 10 | With 90% chance seller earns INR **1667** and buyer earns INR **3333** | OR | With 10% chance seller earns INR **6667** and buyer earns INR **13333** | 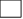 | 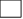 | Seller earns INR **4000** and  buyer earns INR **1000** with 90% chance or INR **16000** with 10% chance |
| 11 | Certainty that seller earns INR **1667** and buyer earns INR **3333** | | | 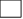 | 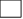 | Certainty that seller earns INR **4000** and  buyer earns INR **1000** |

*[Enumerator should give them enough time and opportunity to discuss and decide. Proceed to next row decision once the previous decision has made firmly]*

You both have taken decisions individually and now you are to take decisions jointly. The procedure for selecting a decision for payment will be explained now.

A randomly selected decision either from the set of individual decisions or from the joint decisions will be paid out. In order to choose whether it is an individual or joint decision, we toss a choice, where if *‘head’* appears, we choose *‘individual decision’* and if *‘tail’* appears, we choose *‘joint decision’* for payment.

If the joint decision is selected for payment, in order to choose a decision from the set of decisions from the decision sheet, you will be asked to choose a card from 11 decks of cards each consists of a number between 1 and 11. The numbers in the card represent the rows in the decision sheet.

If a fixed contract has chosen for the selected row, we will pay Rs. 4000 if it is a seller and if it a buyer we ask to pick a slip from the box consists of a mixture of red and green slips represents the chance of occurrence of high and low price for the corresponding row. If it is a red slip that represented low price earning, the buyer will be paid Rs. 1000 and if it is a green slip represented high price earning, the buyer will be paid Rs. 16000.

And if a shared contract has chosen for the selected row, we ask the agent to pick a slip from the box consists of a mixture of red and green slips which represent the chance of occurrence of high and low price for the corresponding row. If it is a red slip that represented low price earning, Rs. 1667 will be paid if it is a seller and Rs. 3333 if it is a buyer and if it is a green slip that represented high price earning, Rs. 6667 will be paid if it is a seller and Rs, 13333 if it is a buyer.

As we said before there are ……….. number of sellers and ……….. number of buyers in this region to interview and a seller and buyer will be selected randomly for payout. The selection of a buyer and a seller will be done once we finish interviewing above mentioned number of sellers and buyers. The selection is independent of partnership i.e, if a seller got selected for payment does not mean that the buyer for the corresponding seller will also be selected for payment. It could be any buyer and seller. If a seller has more than one joint decision i.e, one for each buyer, any one of the joint decisions will be selected randomly.

If you get selected you will be approached before ………. date. However, you will not know who else got selected for the payment in the region if you not got selected. For now, we do not know the buyer and seller who got selected. Therefore consider your choice seriously.

Do you have any questions

1. **Supplementary tables**

| Table S3: Expected earnings in SC and FC for sellers and buyers for mulberry crop. | | | | | | | | |
| --- | --- | --- | --- | --- | --- | --- | --- | --- |
| Decision row | **Expected earnings in SC** | | **Expected**  **earnings in FC** | | **Diff (SC-FC)** | | **Risk-aversion parameter** | |
|  | **Seller** | **Buyer** | **Seller** | **Buyer** | **Seller** | **Buyer** | **Seller** | **Buyer** |
| 1 | 6667 | 13333 | 4000 | 16000 | 2667 | -2667 | > 3.25 | > 1.15 |
| 2 | 6167 | 12333 | 4000 | 14500 | 2167 | -2167 | 3.25 | 1.15 |
| 3 | 5667 | 11333 | 4000 | 13000 | 1667 | -1667 | 2.18 | 0.76 |
| 4 | 5167 | 10333 | 4000 | 11500 | 1167 | -1167 | 1.44 | 0.49 |
| 5 | 4667 | 9333 | 4000 | 10000 | 667 | -667 | 0.81 | 0.27 |
| 6 | 4167 | 8333 | 4000 | 8500 | 167 | -167 | 0.20 | 0.07 |
| 7 | 3667 | 7333 | 4000 | 7000 | -333 | 333 | -0.42 | -0.14 |
| 8 | 3167 | 6333 | 4000 | 5500 | -833 | 833 | -1.13 | -0.37 |
| 9 | 2667 | 5333 | 4000 | 4000 | -1333 | 1333 | -2.04 | -0.65 |
| 10 | 2167 | 4333 | 4000 | 2500 | -1833 | 1833 | -3.47 | -1.08 |
| 11 | 1667 | 3333 | 4000 | 1000 | -2333 | 2333 | > -3.47 | > -1.08 |

| **Table S4:** Individual decisions faced by buyers and sellers for the maize crop.. | | | | | | | | | | | | |
| --- | --- | --- | --- | --- | --- | --- | --- | --- | --- | --- | --- | --- |
| **Row** | **Buyer decision** | | | | | | **Seller decision** | | | | **Diff. expected earnings**  **(SC-FC)** | |
|  | **SC** | | | **FC** | | | **SC** | | | **FC** | **Buyer** | **Seller** |
| 1 | Certainty of earning INR **6000** | | | Certainty of earning INR **7000** | | | Certainty of earning INR **3000** | | | Certainty of earning INR **2000** | 1000 | -1000 |
| 2 | 10% chance of earning INR **2400** | OR | 90% chance of earning INR **6000** | 10% chance of earning INR **1600** | OR | 90% chance of earning INR **7000** | 10% chance of earning INR **1200** | OR | 90% chance of earning INR **3000** | Certainty of earning INR **2000** | 820 | -820 |
| 3 | 20% chance of earning INR **2400** | OR | 80% chance of earning INR **6000** | 20% chance of earning INR **1600** | OR | 80% chance of earning INR **7000** | 20% chance of earning INR **1200** | OR | 80% chance of earning INR **3000** | Certainty of earning INR **2000** | 640 | -640 |
| 4 | 30% chance of earning INR **2400** | OR | 70% chance of earning INR **6000** | 30% chance of earning INR **1600** | OR | 70% chance of earning INR **7000** | 30% chance of earning INR **1200** | OR | 70% chance of earning INR **3000** | Certainty of earning INR **2000** | 460 | -460 |
|  |  |  |  |  |  |  |  |  |  |  |  |  |
| 5 | 40% chance of earning INR **2400** | OR | 60% chance of earning INR **6000** | 40% chance of earning INR **1600** | OR | 60% chance of earning INR **7000** | 40% chance of earning INR **1200** | OR | 60% chance of earning INR **3000** | Certainty of earning INR **2000** | 280 | -280 |
| 6 | 50% chance of earning INR **2400** | OR | 50% chance of earning INR **6000** | 50% chance of earning INR **1600** | OR | 50% chance of earning INR **7000** | 50% chance of earning INR **1200** | OR | 50% chance of earning INR **3000** | Certainty of earning INR **2000** | 100 | -100 |
| 7 | 60% chance of earning INR **2400** | OR | 40 chance of earning INR **6000** | 60% chance of earning INR **1600** | OR | 40% chance of earning INR **7000** | 60% chance of earning INR **1200** | OR | 40% chance of earning INR **3000** | Certainty of earning INR **2000** | -80 | 80 |
| 8 | 70% chance of earning INR **2400** | OR | 30% chance of earning INR **6000** | 70% chance of earning INR **1600** | OR | 30% chance of earning INR **7000** | 70% chance of earning INR **1200** | OR | 30% chance of earning INR **3000** | Certainty of earning INR **2000** | -260 | 260 |
| 9 | 80% chance of earning INR **2400** | OR | 20% chance earn INR **6000** | 80% chance of earning INR **1600** | OR | 20% chance of earning INR **7000** | 80% chance of earning INR **1200** | OR | 20% chance of earning INR **3000** | Certainty of earning INR **2000** | -440 | 440 |
| 10 | 90% chance of earning INR **2400** | OR | 10% chance of earning INR **6000** | 90% chance of earning INR **1600** | OR | 10% chance of earning INR **7000** | 90% chance of earning INR **1200** | OR | 10% chance of earning INR **3000** | Certainty of earning INR **2000** | -620 | 620 |
| 11 | Certainty of earning INR **2400** | | | Certainty of earning INR **1600** | | | Certainty of earning INR **1200** | | | Certainty of earning INR **2000** | -800 | 800 |

| **Table S5:** Joint decisions faced by sellers and buyers for maize crop. | | | | | | |
| --- | --- | --- | --- | --- | --- | --- |
| **Decision row** | **Earnings in SC** | | | **Your choice** | | **Earnings in FC** |
|  |  |  |  | SC | FC |  |
| 1 | Certainty that seller earns INR **3000** and  buyer earns INR **6000** | | |  |  | Certainty that seller earns INR **2000** and  buyer earns INR **7000** |
| 2 | With 10% chance seller earns INR **1200** and buyer earns INR **2400** | OR | With 90% chance seller earns INR **3000** and buyer earns INR **6000** |  |  | Seller earns INR **2000** and  buyer earns INR **1600** with 10% chance or INR **7000** with 90% chance |
| 3 | With 20% chance seller earns INR **1200** and buyer earns INR **2400** | OR | With 80% chance seller earns INR **3000** and buyer earns INR **6000** |  |  | Seller earns INR **2000** and  buyer earns INR **1600** with 20% chance or INR **7000** with 80% chance |
| 4 | With 30% chance seller earns INR **1200** and buyer earns INR **2400** | OR | With 70% chance seller earns INR **3000** and buyer earns INR **6000** |  |  | Seller earns INR **2000** and  buyer earns INR **1600** with 30% chance or INR **7000** with 70% chance |
| 5 | With 40% chance seller earns INR **1200** and buyer earns INR **2400** | OR | With 60% chance seller earns INR **3000** and buyer earns INR **6000** |  |  | Seller earns INR **2000** and  buyer earns INR **1600** with 40% chance or INR **7000** with 60% chance |
| 6 | With 50% chance seller earns INR **1200** and buyer earns INR **2400** | OR | With 50% chance seller earns INR **3000** and buyer earns INR **6000** |  |  | Seller earns INR **2000** and  buyer earns INR **1600** with 50% chance or INR **7000** with 50% chance |
| 7 | With 60% chance seller earns INR **1200** and buyer earns INR **2400** | OR | With 40% chance seller earns INR **3000** and buyer earns INR **6000** |  |  | Seller earns INR **2000** and  buyer earns INR **1600**with 60% chance or INR **7000** with 40% chance |
| 8 | With 70% chance seller earns INR **1200** and buyer earns INR **2400** | OR | With 30% chance seller earns INR **3000** and buyer earns INR **6000** |  |  | Seller earns INR **2000** and  buyer earns INR **1600** with 70% chance or INR **7000** with 30% chance |
| 9 | With 80% chance seller earns INR **1200** and buyer earns INR **2400** | OR | With 20% chance seller earns INR **3000** and buyer earns INR **6000** |  |  | Seller earns INR **2000** and  buyer earns INR **1600** with 80% chance or INR **7000** with 20% chance |
| 10 | With 90% chance seller earns INR **1200** and buyer earns INR **2400** | OR | With 10% chance seller earns INR **3000** and buyer earns INR **6000** |  |  | Seller earns INR **2000** and  buyer earns INR **1600** with 90% chance or INR **7000** with 10% chance |
| 11 | Certainty that seller earns INR **1200** and buyer earns INR **2400** | | |  |  | Certainty that seller earns INR **2000** and  buyer earns INR **1600** |

| Table S6: Expected earnings in SC and FC for sellers and buyers for maize crop. | | | | | | | | |
| --- | --- | --- | --- | --- | --- | --- | --- | --- |
| Decision row | **Expected earnings in SC** | | **Expected earnings in FC** | | **Diff (SC-FC)** | | **Risk-aversion parameter** | |
|  | Seller | Buyer | Seller | Buyer | Seller | Buyer | Seller | Buyer |
| 1 | 3000 | 6000 | 2000 | 7000 | 1000 | -1000 | > 5.15 | > 2.02 |
| 2 | 2820 | 5640 | 2000 | 6460 | 820 | -820 | 5.15 | 2.02 |
| 3 | 2640 | 5280 | 2000 | 5920 | 640 | -640 | 3.47 | 1.35 |
| 4 | 2460 | 4920 | 2000 | 5380 | 460 | -460 | 2.33 | 0.90 |
| 5 | 2280 | 4560 | 2000 | 4840 | 280 | -280 | 1.38 | 0.53 |
| 6 | 2100 | 4200 | 2000 | 4300 | 100 | -100 | 0.49 | 0.19 |
| 7 | 1920 | 3840 | 2000 | 3760 | -80 | 80 | -0.41 | -0.15 |
| 8 | 1740 | 3480 | 2000 | 3220 | -260 | 260 | -1.40 | -0.53 |
| 9 | 1560 | 3120 | 2000 | 2680 | -440 | 440 | -2.64 | -0.99 |
| 10 | 1380 | 2760 | 2000 | 2140 | -620 | 620 | -4.54 | -1.68 |
| 11 | 1200 | 2400 | 2000 | 1600 | -800 | 800 | > -4.54 | > -1.68 |

| **Table S7:** Individual decisions faced by buyers and sellers for the Chrysanthemum crop. | | | | | | | | | | | | |
| --- | --- | --- | --- | --- | --- | --- | --- | --- | --- | --- | --- | --- |
| **Row** | **Buyer decision** | | | | | | **Seller decision** | | | | **Diff. expected earnings**  **(SC-FC)** | |
|  | **SC** | | | **FC** | | | **SC** | | | **FC** | **Buyer** | **Seller** |
| 1 | Certainty of earning INR **38000** | | | Certainty of earning INR **47000** | | | Certainty of earning INR **19000** | | | Certainty of earning INR**10000** | 9000 | -9000 |
| 2 | 10% chance of earning INR **6800** | OR | 90% chance of earning INR **38000** | 10% chance of earning INR **200** | OR | 90% chance of earning INR **47000** | 10% chance of earning INR **3400** | OR | 90% chance of earning INR **19000** | Certainty of earning INR**10000** | 7440 | -7440 |
| 3 | 20% chance of earning INR **6800** | OR | 80% chance of earning INR **38000** | 20% chance of earning INR **200** | OR | 80% chance of earning INR **47000** | 20% chance of earning INR **3400** | OR | 80% chance of earning INR **19000** | Certainty of earning INR**10000** | 5880 | -5880 |
| 4 | 30% chance of earning INR **6800** | OR | 70% chance of earning INR **38000** | 30% chance of earning INR **200** | OR | 70% chance of earning INR **47000** | 30% chance of earning INR **3400** | OR | 70% chance of earning INR **19000** | Certainty of earning INR**10000** | 4320 | -4320 |
|  |  |  |  |  |  |  |  |  |  |  |  |  |
| 5 | 40% chance of earning INR **6800** | OR | 60% chance of earning INR **38000** | 40% chance of earning INR **200** | OR | 60% chance of earning INR **47000** | 40% chance of earning INR **3400** | OR | 60% chance of earning INR **19000** | Certainty of earning INR**10000** | 2760 | -2760 |
| 6 | 50% chance of earning INR **6800** | OR | 50% chance of earning INR **38000** | 50% chance of earning INR **200** | OR | 50% chance of earning INR **47000** | 50% chance of earning INR **3400** | OR | 50% chance of earning INR **19000** | Certainty of earning INR **2000** | 100 | -100 |
| 7 | 60% chance of earning INR **6800** | OR | 40 chance of earning INR **38000** | 60% chance of earning INR **200** | OR | 40% chance of earning INR **47000** | 60% chance of earning INR **3400** | OR | 40% chance of earning INR **19000** | Certainty of earning INR**10000** | -80 | 80 |
| 8 | 70% chance of earning INR **6800** | OR | 30% chance of earning INR **38000** | 70% chance of earning INR **200** | OR | 30% chance of earning INR **47000** | 70% chance of earning INR **3400** | OR | 30% chance of earning INR **19000** | Certainty of earning INR**10000** | -1920 | 1920 |
| 9 | 80% chance of earning INR **6800** | OR | 20% chance earn INR **38000** | 80% chance of earning INR **200** | OR | 20% chance of earning INR **47000** | 80% chance of earning INR **3400** | OR | 20% chance of earning INR **19000** | Certainty of earning INR**10000** | -3480 | 3480 |
| 10 | 90% chance of earning INR **6800** | OR | 10% chance of earning INR **38000** | 90% chance of earning INR **1600** | OR | 10% chance of earning INR **47000** | 90% chance of earning INR **3400** | OR | 10% chance of earning INR **19000** | Certainty of earning INR**10000** | -5040 | 5040 |
| 11 | Certainty of earning INR **6800** | | | Certainty of earning INR **1600** | | | Certainty of earning INR **3400** | | | Certainty of earning INR**10000** | -6600 | 6600 |

| **Table S8:** Joint decisions faced by sellers and buyers for chrysanthemum crop. | | | | | | |
| --- | --- | --- | --- | --- | --- | --- |
| **Decision row** | **Earnings in SC** | | | **Your choice** | | **Earnings in FC** |
| 1 | Certainty that seller earns INR **19000** and buyer earns INR **38000** | | | SC | FC | Certainty that seller earns INR **10000** and buyer earns INR **47000** |
| 2 | With 10% chance seller earns INR **3400** and buyer earns INR **6800** | OR | With 90% chance seller earns INR **19000** and buyer earns INR **38000** |  |  | Seller earns INR **10000** and buyer earns INR **200** with 10% chance or INR **47000** with 90% chance |
| 3 | With 20% chance seller earns INR **3400** and buyer earns INR **6800** | OR | With 80% chance seller earns INR **19000** and buyer earns INR **38000** |  |  | Seller earns INR **10000** and buyer earns INR **200** with 20% chance or INR **47000** with 80% chance |
| 4 | With 30% chance seller earns INR **3400** and buyer earns INR **6800** | OR | With 70% chance seller earns INR **19000** and buyer earns INR **38000** |  |  | Seller earns INR **10000** and buyer earns INR **200** with 30% chance or INR **47000**with 70% chance |
| 5 | With 40% chance seller earns INR **3400** and buyer earns INR **6800** | OR | With 60% chance seller earns INR **19000** and buyer earns INR **38000** |  |  | Seller earns INR **10000** and buyer earns INR **200** with 40% chance or INR **47000** with 60% chance |
| 6 | With 50% chance seller earns INR **3400**  and, buyer earns INR **6800** | OR | With 50% chance seller earns INR **19000** and buyer earns INR **38000** |  |  | Seller earns INR **10000** and buyer earns INR **200** with 50% chance or INR **47000** with 50% chance |
| 7 | With 60% chance seller earns INR **3400** and buyer earns INR **6800** | OR | With 40% chance seller earns INR **19000** and buyer earns INR **38000** |  |  | Seller earns INR **10000** and buyer earns INR **200** with 60% chance or INR **47000** with 40% chance |
| 8 | With 70% chance seller earns INR **3400** and buyer earns INR **6800** | OR | With 30% chance seller earns INR **19000** and buyer earns INR **38000** |  |  | Seller earns INR **10000** and buyer earns INR **200** with 70% chance or INR **47000** with 30% chance |
| 9 | With 80% chance seller earns INR **3400** and buyer earns INR **6800** | OR | With 20% chance seller earns INR **19000** and buyer earns INR **38000** |  |  | Seller earns INR **10000** and buyer earns INR **200** with 80% chance or INR **47000** with 20% chance |
| 10 | With 90% chance seller earns INR **3400** and buyer earns INR **6800** | OR | With 10% chance  seller earns INR **19000** and buyer earns INR **38000** |  |  | Seller earns INR **10000** and buyer earns INR **200** with 90% chance or INR **47000** with 10% chance |
| 11 | Certainty that seller earns INR **3400** and buyer earns INR **6800** | | |  |  | Certainty that seller earns INR **10000** and buyer earns INR **200** |

| Table S9: Expected earnings in SC and FC for sellers and buyers for chrysanthemum crop. | | | | | | | | |
| --- | --- | --- | --- | --- | --- | --- | --- | --- |
| Decision row | **Expected earnings in SC** | | **Expected earnings in FC** | | **Diff (SC-FC)** | | **Risk-aversion parameter** | |
|  | Seller | Buyer | Seller | Buyer | Seller | Buyer | Seller | Buyer |
| 1 | 19000 | 38000 | 10000 | 47000 | 9000 | -9000 | > 2.83 | > 0.83 |
| 2 | 17440 | 34880 | 10000 | 42320 | 7440 | -7440 | 2.83 | 0.83 |
| 3 | 15880 | 31760 | 10000 | 37640 | 5880 | -5880 | 1.97 | 0.58 |
| 4 | 14320 | 28640 | 10000 | 32960 | 4320 | -4320 | 1.38 | 0.40 |
| 5 | 12760 | 25520 | 10000 | 28280 | 2760 | -2760 | 0.87 | 0.25 |
| 6 | 11200 | 22400 | 10000 | 23600 | 100 | -100 | 0.38 | 0.11 |
| 7 | 9640 | 19280 | 10000 | 18920 | -80 | 80 | -0.12 | -0.04 |
| 8 | 8080 | 16160 | 10000 | 14240 | -1920 | 1920 | -0.69 | -0.20 |
| 9 | 6520 | 13040 | 10000 | 9560 | -3480 | 3480 | -1.41 | -0.41 |
| 10 | 4960 | 9920 | 10000 | 4880 | -5040 | 5040 | -2.56 | -0.74 |
| 11 | 3400 | 6800 | 10000 | 200 | -6600 | 6600 | > -2.56 | > -0.74 |
