# Supplementary material for: Do buyers have bargaining power? Evidence from informal groundwater contracts
Source: PLoS One. 2020 Sep 30;15(9):e0236696. doi: 10.1371/journal.pone.0236696 (PMC7527252; doi:10.1371/journal.pone.0236696)
Supplement: S1 Appendix — (DOCX) [file pone.0236696.s003.docx]

**Appendix**

| **Table A1:** Water use, water price, and profitability in SC and FC contracts. | | | | | | | |
| --- | --- | --- | --- | --- | --- | --- | --- |
| **Particulars** | **Mulberry** | | | **Tomato** | | **Maize** | |
|  | SC | | FC | SC | FC | SC | FC |
| No. of Obs. | 11 | | 4 | 6 | 8 | 31 | 2 |
| Water price per acre per season (INR) | 10,400  (4200) | | 6,000  (3000) | 13,000  (9300) | 11,000  (5800) | 4,400  (1400) | 3,900  (800) |
| Water used per acre pre season (in gallons) | 221,000  (103000) | | 186,000  (52000) | 494,000  (265000) | 462,000  (152000) | 751,000  (421000) | 743,000  (385000) |
| Price per hr of pumping (INR) | 134  (40) | | 84  (37) | 84  (42) | 83  (80) | 23  (7) | 28  (7) |
| Yield per acre (kgs) | 143  (36) | | 213  (25) | 7,400  (1740) | 9,200  (2900) | 1,000  (240) | 1,600  (71) |
| Output Price per kg (INR) | 239  (51) | | 299  (7) | 6  (3) | 12  (11) | 13  (2) | 12  (2) |
| Total cost per acre (INR) | 19,900  (9100) | | 19,800  (16200) | 31,100  (7800) | 50,400  (26600) | 18,800  (6000) | 15,800  (3200) |
| *Net profit per acre (INR)* | | | | | | | |
| Mean | | 4,300  (12000) | 37,800  (10000) | 2,500  (24200) | 42,000  (60700) | -9,400  (5800) | -3,300  (5400) |
| Median | | 2,400 | 37,300 | -5,200 | 49,500 | -8,500 | -3,300 |
| *1 gallon = 3.7 liters*  *Standard deviation in parentheses* | | | | | | | |

**<<Figure A1**: Degree of disagreement between buyer’s and seller’s preferences>>

| **Table A2: Joint decisions faced by sellers and buyers for the mulberry crop.** **Seller ID ……………… , Buyer ID ……………** | | | | | | |
| --- | --- | --- | --- | --- | --- | --- |
| **Decision row** | **Earnings in SC** | | | **Your choice** | | **Earnings in FC** |
|  |  |  |  | **SC** | **FC** |  |
| 1 | Certainty that seller earns INR **6667** and buyer earns INR **13333** | | | 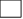 | 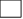 | Certainty that seller earns INR **4000** and buyer earns INR **16000** |
| 2 | With 10% chance seller earns INR **1667** and buyer earns INR **3333** | OR | With 90% chance seller earns INR **6667** and buyer earns INR **13333** | 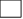 | 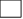 | Seller earns INR **4000** and buyer earns INR **1000** with 10% chance or INR **16000** with 90% chance |
| 3 | With 20% chance seller earns INR **1667** and buyer earns INR **3333** | OR | With 80% chance seller earns INR **6667** and buyer earns INR **13333** | 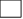 | 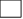 | Seller earns INR **4000** and  buyer earns INR **1000** with 20% chance or INR **16000** with 80% chance |
| 4 | With 30% chance seller earns INR **1667** and buyer earns INR **3333** | OR | With 70% chance seller earns INR **6667** and buyer earns INR **13333** | 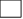 | 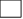 | Seller earns INR **4000** and  buyer earns INR **1000** with 30% chance or INR **16000** with 70% chance |
| 5 | With 40% chance seller earns INR **1667** and buyer earns INR **3333** | OR | With 60% chance seller earns INR **6667** and buyer earns INR **13333** | 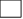 | 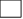 | Seller earns INR **4000** and  buyer earns INR **1000** with 40% chance or INR **16000** with 60% chance |
| 6 | With 50% chance seller earns INR **1667** and buyer earns INR **3333** | OR | With 50% chance seller earns INR **6667** and buyer earns INR **13333** | 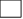 | 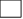 | Seller earns INR **4000** and  buyer earns INR **1000** with 50% chance or INR **16000** with 50% chance |
| 7 | With 60% chance seller earns INR **1667** and buyer earns INR **3333** | OR | With 40% chance seller earns INR **6667** and buyer earns INR **13333** | 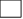 | 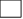 | Seller earns INR **4000** and  buyer earns INR **1000** with 60% chance or INR **16000** with 40% chance |
| 8 | With 70% chance seller earns INR **1667** and buyer earns INR **3333** | OR | With 30% chance seller earns INR **6667** and buyer earns INR **13333** | 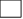 | 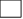 | Seller earns INR **4000** and  buyer earns INR **1000** with 70% chance or INR **16000** with 30% chance |
| 9 | With 80% chance seller earns INR **1667** and buyer earns INR **3333** | OR | With 20% chance seller earns INR **6667** and buyer earns INR **13333** | 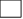 | 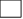 | Seller earns INR **4000** and  buyer earns INR **1000** with 80% chance or INR **16000** with 20% chance |
| 10 | With 90% chance seller earns INR **1667** and buyer earns INR **3333** | OR | With 10% chance seller earns INR **6667** and buyer earns INR **13333** | 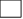 | 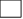 | Seller earns INR **4000** and  buyer earns INR **1000** with 90% chance or INR **16000** with 10% chance |
| 11 | Certainty that seller earns INR **1667** and buyer earns INR **3333** | | | 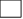 | 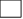 | Certainty that seller earns INR **4000** and  buyer earns INR **1000** |

| **Table A3**: Variable description | | |
| --- | --- | --- |
| **SL no.** | **Variable name** | **Description** |
| *I.* | *Dependent variables* |  |
| 1 | Choice of SC | Binary variable taking the value 1 if the agent chooses output-shared contract in the individual decision series, and 0 others wise. |
| 2 | Joint decision=buyers preference | Binary variable taking the value 1 if the joint decision corresponds to buyer's individual preference, and 0 others wise. |
| *II.* | *Independent variables* |  |
| 3 | Difference in earnings (SC – FC) if SC> FC | Continuous variable measured as a difference in the earning between SC and FC when SC earning is higher than FC |
| 4 | Difference in earnings (SC – FC) if SC< FC | Continuous variable measured as a difference in the earning between SC and FC when SC earning is lower than FC |
| 5 | Degree of disagreement  \| Prob^b^(sc) – Prob^s^(sc) \| | Preference divergence between buyers and sellers. It is measured as a score varies from 0 to 1. Value 0 indicates buyer and seller have a similar preference and 1 indicates both have a contrary preference |
| 6 | Kinship ties | Binary variable taking the value 1 if water sellers and buyers pair share kin relationship, and 0 others wise. |
| 7 | Education | Continuous variable measuring the number of schooling years |
| 8 | Landholdings | Continuous variable measuring the number of acres of land owned |
| 9 | Previous contract: SC | Binary variable taking the value 1 sellers and buyers pair had SC during the baseline survey, and 0 others wise |
| 10 | Previous contract: Other than SC | Binary variable taking the value 1 sellers and buyers pair other than SC during the baseline survey, and 0 others wise. |
| 11 | No. of potential sellers | Continuous variable measuring the number of additional potential sellers that the buyers had in the deliverable area |
| *III.* | *Buyers characteristics relative to sellers* | |
| 12 | Buyer owns more land than the seller | Binary variable taking the value 1 buyer owns more land than sellers, and 0 others wise |
| 13 | Buyer more education than the seller | Binary variable taking the value 1 buyer is more educated than sellers, and 0 others wise |
| 14 | Buyer older than the seller | Binary variable taking the value 1 buyer is older than sellers, and 0 others wise |
|  | *Crop dummies* |  |
| 15 | Crop: Mulberry | Dummy variable taking the value 1 if the crop is mulberry, and 0 others wise |
| 16 | Crop: Maize | Dummy variable taking the value 1 if the crop is Maize, and 0 others wise |

| **Table A4**: Marginal effects of multinomial probit model with 4 categories of choices. | | | | |
| --- | --- | --- | --- | --- |
| **Variables** | **(1)** | **(2)** | **(3)** | **(4)** |
|  | **Joint=Seller** | **Joint=Buyer** | **Joint=Seller=Buyer** | **Choice shift case** |
| Degree of Disagreement  \| Prob^b^(sc) – Prob^s^(sc) \| | 0.476*** | 0.152*** | -0.385*** | -0.242*** |
|  | (0.046) | (0.047) | (0.031) | (0.026) |
| Kinship ties | -0.131*** | 0.138*** | 0.004 | -0.011 |
|  | (0.035) | (0.036) | (0.024) | (0.019) |
| Years of contract | -0.011** | 0.007 | 0.008** | -0.003 |
|  | (0.006) | (0.005) | (0.003) | (0.003) |
| Previous contract: SC | 0.071 | -0.083 | -0.029 | 0.041 |
|  | (0.065) | (0.055) | (0.048) | (0.034) |
| No. of potential sellers | -0.020 | 0.071* | 0.046 | -0.096** |
|  | (0.055) | (0.040) | (0.033) | (0.049) |
| Buyer owns more land than seller | -0.015 | -0.010 | 0.039 | -0.014 |
|  | (0.041) | (0.039) | (0.027) | (0.023) |
| Buyer more education than seller | -0.040 | 0.029 | 0.010 | 0.002 |
|  | (0.039) | (0.037) | (0.026) | (0.019) |
| Buyer older than seller | 0.057 | -0.033 | 0.007 | -0.032 |
|  | (0.042) | (0.037) | (0.028) | (0.021) |
| Crop: Mulberry | 0.094 | -0.093* | -0.001 | -0.001 |
|  | (0.059) | (0.050) | (0.043) | (0.030) |
| Crop: Maize | 0.190*** | -0.109*** | -0.066*** | -0.015 |
|  | (0.042) | (0.041) | (0.024) | (0.022) |
| No. of observations | 1,947 | 1,947 | 1,947 | 1,947 |
| No. of pairs | 177 | 177 | 177 | 177 |
| *Standard errors in parentheses, *** p<0.01, ** p<0.05, * p<0.1* | | | | |

**Figure A1**: Degree of disagreement between buyer’s and seller’s preferences
