# Supplementary material for: Do buyers have bargaining power? Evidence from informal groundwater contracts
Source: PLoS One. 2020 Sep 30;15(9):e0236696. doi: 10.1371/journal.pone.0236696 (PMC7527252; doi:10.1371/journal.pone.0236696)
Supplement: Attachment — Submitted filename: Review PONE-D-19-33331 Dec 27 2019.pdf [file pone.0236696.s004.pdf]

Do buyers have bargaining power? Evidence from informal groundwater contracts

Reviewer's Comments:

The paper "Do buyers have bargaining power? Evidence from informal groundwater contracts", aims to examine the relative bargaining power of sellers and buyers in informal groundwater markets in India. This author has spent significant efforts in designing a framed-field experiment and using probit regression model to explain the preferences of sellers and buyers and their joint decisions. More importantly, this research has unique data set which consists of actual sellers and buyers in groundwater contracts. This paper has potential to become a paper with great contributions to literature and practitioners. Despite the excellent methodology and data, to be considered for publication in a highly regarded journal, the study needs to address some key issues with the current version. Therefore, my recommendation is "major revision" toward the current version of manuscript.

The major issues are summarized below.

1. From the title, "Do buyers have bargaining power?", it seems that this paper aims to do research about the bargaining power. However, the author conducted almost no literature review about bargaining power, leading to a significant flaw of this paper. What have we known about buyer's bargaining power from previous studies? What factors will lead to more power possessed by buyers? Without thorough literature review, it is difficult to convince readers that the model is correctly developed to measure bargaining power and control relevant variables. I strongly suggest that the author use resource-based view (RBV) and resource dependency theory (RDT) as overarching theories to conduct this study. After Section 1 Introduction, there should be Section 2 Literature Review, which covers the literature related to bargaining power and relevant overarching theories.
2. The second issue is relevant to item 1. The author should propose strong reasoning for including socioeconomic characteristics such as education, landholdings, and previous contract in the model. If the author wants to include these variables in the model, there must be strong reasons supported by previous studies and, at least, a paragraph to explain the logic and prediction of sign of regression parameters.
3. The third issue is, again, relevant to item 1. The contribution of this paper is not clear. Because of the lack in literature review on bargaining power, the author proposes neither research gap in the bargaining power nor hypotheses. It seems that the author is more familiar with the groundwater market studies, which constrain the generalizability of this study. Hence, whether the findings of this study can be generalized to other context becomes a doubt. If the findings cannot be generalized, the contribution of this paper will be limited.

Some minor issues are found as follows.

1. The writing of this paper can be further enhanced. Because most readers may not be familiar with different types of contracts and groundwater market studies, the author should explain the terms when they are mentioned for the first time. For example, there should be explanation about SC and FC in Section 1 or 2.

2. The measurement of each variable should be explained clearly. For example, I am not sure how the author measure education and kin relationship until I read through the result section. I am not sure whether kinship tie is a dummy variable even after I see the result in Section 5.3.
3. The equations do not match the result. For example, Equation 3 does not include socioeconomic characteristics as shown in Table 5.
4. In the second sentence of Section 5.3, the significance level for the coefficient of the degree of disagreement should be 1 percent, not 10 percent.
