# Supplementary material for: Do buyers have bargaining power? Evidence from informal groundwater contracts
Source: PLoS One. 2020 Sep 30;15(9):e0236696. doi: 10.1371/journal.pone.0236696 (PMC7527252; doi:10.1371/journal.pone.0236696)
Supplement: Attachment — Submitted filename: Response letter_Reviewer 2.docx [file pone.0236696.s005.docx]

**Letter of changes for Reviewer 2:**

# *Manuscript title- Do buyers have bargaining power? Evidence from informal groundwater contracts*

**Summary of changes**

1. The introduction has been significantly re-written according to the reviewer’s comments.
2. A review section (section 2) has been added as for the reviewer’s recommendation.
3. The major part of the conceptual framework has been re-oriented to emphasize bargaining power and rent extraction in contractual agreements.
4. The model section has been added with more information on the socio-economics aspect and a prior expectation related to each of them.
5. In the results section, risk orientation has been removed and added more discussion on the results.

**Details of changes made corresponding to the comment**

Thank you very much for the comments. I am glad about the meticulous review of the paper. Below is my response to each of the comments.

This is a very interesting research topic and an empirical study.

But there is no sufficient literature review, which leads to several flaws decreasing the quality of the paper:

1. The concept of bargaining power used in the paper is not clearly defined and operationalized. There is an extensive literature on bargaining power and different approaches are chosen to measure power relations. Quantitative works most often refer to farm size (quantities produced), the distance between contractors, and farmers’ outside options. Farmers’ bargaining power may also stem from other things – such as the quality of a farm’s output and qualitative factors such as having personal contacts, the length of relationships, negotiating skills, etc. Therefore, a choice of variables (e.g. in equation 4) should be explained. For example why kinship ties should be included in the analysis? It is not explained. A large part of the analysis is related to risk issues. How does this relate to bargaining power?

Answer comment 1:

Thanks for a very in-depth reading of the manuscript. I agree with your comments; the study should be more focused on bargaining. Now a few sections of the manuscript have been significantly re-written. Here I mention those changes.

a) Re-orientation of introduction:

The introduction has been significantly re-oriented towards bargaining. In paragraph 3 I discussed how previous literature in the agricultural markets defined power balance between the agent, how hedonic pricing models were used to estimate bargaining power linked with agents’ socio-economic characteristics and how it is not straight forward to use hedonic model (price is a function of attributes and agent’s characteristics) when it comes to agricultural contract due to unique characteristics of informal agrarian markets. In paragraph 4, I explain what I do it in this study, and in paragraph 5, I detail the unique contribution of the study by reflecting on the previous studies' limitations.

b) Addition of review section:

The new section 2 named ‘Bargaining in agrarian contracts’ has been added which gives a literature review on bargaining in agricultural contracts. This section starts with how hedonic pricing models were used in determining sellers and buyers supply and demand prices in the market. Second passage describes how hedonic pricing models were commonly used to estimate the excess surplus in the market by deriving buyers' willingness to pay, and sellers willing to accept for a product. In the third paragraph, I describe how the hedonic pricing model was modified to account for and explain the bargaining power of agents in the housing market. How these models were later adopted in agricultural markets such as land-rental and water market and general findings were described in passage 4.

c) Model section:

Now I have added an explanation about the regressors which I used and detailed expected effects for each of the regressor in both section 5.1 and 5.2. I have also added Appendix table A3 that describes how I measure the variables used in the analysis.

d) I agree that risk aversion explanations were not fitting well here. Now I have removed the passage on risk aversion behaviour of the sellers and buyer.

2. My impression is also that some concepts are confused (e.g. market power and bargaining power). Sometimes unclear assumptions are also presented. For example, the Author writes on page 8, "Each agent has some power to influence the outcome in his or her favour and the final choice of the contract depends on who has more bargaining power to influence the joint decision." And on the next page, "We hypothesise, therefore, that sellers and buyers have equal bargaining power in contract decisions."

Answer comment 2:

Thanks for this very important comment. I kind of overlooked to provide the right distinction and the emphasis of the study.

- To bring clarity early enough, In the introduction section, I have now added a footnote 4 where I mention *“The confusion arises then about the notion of bargaining power and market power. Technically, bargaining power and market power results in the extraction of surplus, however, the concept of bargaining power is more appropriate in this study. More discussion on section 4.2”*
- In section 4.2, now I have included a paragraph discussing the difference between bargaining power and market power. I mention *“Although, in principle bargaining power and market power may result in extraction or transfer of surplus, I believe that the bargaining power is appropriate in the unique case of informal agrarian markets. In the bargaining power, market participant exerts power to obtain concession or extract surplus from another party using the threat of contractual break, while the market power is the ability to lower the supply and sustain demand by placing price about cost (Jan Svejnar, 1986, Sorrentino et al, 2018). Further bargaining framework incorporate cooperation and coordination agreements, while market power models are non-cooperative in nature (Sorrentino et al, 2018)”*

3. A lack of sound conceptual description leaves a reader with the impression that the paper investigates contract choices rather than bargaining power.

Answer comment 2:

Thanks for this constructive comment. I realized that readers are missing the thread of why the author used contract choice instead of the price of water to elicit the bargaining power of agents was missing.

Most studies in the agricultural contract either used price mark-up or hedonic pricing models remarks on agents' bargaining power. The Price mark-up is a very naïve way to estimate bargaining power. Later studies adopted hedonic pricing models where the price is a function of product attributes and other agent’s characteristics. The coefficients of the agent’s characteristics were used as an indicator of the ability of agents to push-up or pull-down the prices. These bargaining models were used in the housing market mainly where agents negotiate on “price” alone.

In the case of agricultural contracts, agents negotiate on the contract type before the price where different contract gives different flexibility to agents regarding the payment and risk-sharing. For example, in the output-shared contract, the buyer pays a price at the end of the season as a fixed share of total output produced, while in fixed-price contract buyer pays the fixed amount at the start of the season in terms of cash. Given this structural difference across contractual type, agents make decisions sequentially, first, they decide on the choice of contract and then the price is negotiated. In most cases, the price is a consequential outcome of the contract type that the agents choose. Therefore, the use of hedonic price modeling in the agricultural contracts leads to a biased estimation of agents' bargaining power, because the personal characteristics of agents affect both prices as well as preferences for contract type. Thus I chose agent negotiation instead of price to avoid the simultaneity bias.

This was missing in the previous manuscript. Now I have added the above explanation in the introduction. I hope now it makes it clear why this study deviates from price function models to contract function model.

4. Empirical studies on water markets as well as a description of the specific situation in India are not well described. For example, the data on the water market in India comes from 1998 (p.2). If there are no new estimates, it should be stressed.

Answer comment 4:

Thanks for the comments. In the introduction paragraph 1 and 2 mainly describe the situation in water markets in developing countries. In section 3, I have now added a few more details and cited some recent estimates specific to India. However, there are no recent comprehensive recent estimates for the country, as most studies on groundwater are specific to states or a geographic location. There is no nationwide study yet.

5. There is no good discussion of results (it is included in the conclusions section).

Answer comment 5:

Thanks for the comments. As you mentioned, in the results section I mainly explained and interpreted the implications of the findings, keeping the most discussion in the conclusions. I believed that it helps the reader to know the results at once.

In the revised manuscript, I have moved some of the discussion into the results (see section 6.2, and 6.3).

6. Due to the above flaws, the implications presented in conclusions do not seem sound and convincing.

Answer comment 6:

Since most sections like introduction, literature review, conceptual framework, the model, and results are significantly re-written. I hope now the result and conclusions are more convincing. I am open to any specific suggestions!
