# Supplementary material for: Do buyers have bargaining power? Evidence from informal groundwater contracts
Source: PLoS One. 2020 Sep 30;15(9):e0236696. doi: 10.1371/journal.pone.0236696 (PMC7527252; doi:10.1371/journal.pone.0236696)
Supplement: Attachment — Submitted filename: Editor and reviwer comments.docx [file pone.0236696.s006.docx]

**PONE-D-19-33331R1
Do buyers have bargaining power? Evidence from informal groundwater contracts
PLOS ONE**

Dear Dr. Yashodha,

Thank you for submitting your manuscript to PLOS ONE. After careful consideration, we feel that it has merit but does not fully meet PLOS ONE’s publication criteria as it currently stands. Therefore, we invite you to submit a revised version of the manuscript that addresses the points raised during the review process.

I recommend that it should be revised taking into account the changes requested by Reviewers. I would like to give you the last chance to revise your manuscript. To speed the review process, the manuscript will only be reviewed by the Academic Editor in the next round.

Please submit your revised manuscript by Jul 23 2020 11:59PM. If you will need more time than this to complete your revisions, please reply to this message or contact the journal office at plosone@plos.org. When you're ready to submit your revision, log on to https://www.editorialmanager.com/pone/ and select the 'Submissions Needing Revision' folder to locate your manuscript file.

Please include the following items when submitting your revised manuscript:

- A rebuttal letter that responds to each point raised by the academic editor and reviewer(s). You should upload this letter as a separate file labeled 'Response to Reviewers'.
- A marked-up copy of your manuscript that highlights changes made to the original version. You should upload this as a separate file labeled 'Revised Manuscript with Track Changes'.
- An unmarked version of your revised paper without tracked changes. You should upload this as a separate file labeled 'Manuscript'.

If you would like to make changes to your financial disclosure, please include your updated statement in your cover letter. Guidelines for resubmitting your figure files are available below the reviewer comments at the end of this letter.

If applicable, we recommend that you deposit your laboratory protocols in protocols.io to enhance the reproducibility of your results. Protocols.io assigns your protocol its own identifier (DOI) so that it can be cited independently in the future. For instructions see: <http://journals.plos.org/plosone/s/submission-guidelines#loc-laboratory-protocols>

We look forward to receiving your revised manuscript.

Kind regards,

Baogui Xin, Ph.D.
Academic Editor
PLOS ONE


[Note: HTML markup is below. Please do not edit.]

Reviewers' comments:

Reviewer's Responses to Questions

**Comments to the Author**

1. If the authors have adequately addressed your comments raised in a previous round of review and you feel that this manuscript is now acceptable for publication, you may indicate that here to bypass the “Comments to the Author” section, enter your conflict of interest statement in the “Confidential to Editor” section, and submit your "Accept" recommendation.

Reviewer #1: All comments have been addressed

Reviewer #2: (No Response)

2. Is the manuscript technically sound, and do the data support the conclusions?

The manuscript must describe a technically sound piece of scientific research with data that supports the conclusions. Experiments must have been conducted rigorously, with appropriate controls, replication, and sample sizes. The conclusions must be drawn appropriately based on the data presented.

Reviewer #1: Yes

Reviewer #2: Yes

3. Has the statistical analysis been performed appropriately and rigorously?

Reviewer #1: Yes

Reviewer #2: Yes

4. Have the authors made all data underlying the findings in their manuscript fully available?

The [PLOS Data policy](http://www.plosone.org/static/policies.action#sharing) requires authors to make all data underlying the findings described in their manuscript fully available without restriction, with rare exception (please refer to the Data Availability Statement in the manuscript PDF file). The data should be provided as part of the manuscript or its supporting information, or deposited to a public repository. For example, in addition to summary statistics, the data points behind means, medians and variance measures should be available. If there are restrictions on publicly sharing data—e.g. participant privacy or use of data from a third party—those must be specified.

Reviewer #1: Yes

Reviewer #2: Yes

5. Is the manuscript presented in an intelligible fashion and written in standard English?

PLOS ONE does not copyedit accepted manuscripts, so the language in submitted articles must be clear, correct, and unambiguous. Any typographical or grammatical errors should be corrected at revision, so please note any specific errors here.

Reviewer #1: Yes

Reviewer #2: (No Response)

6. Review Comments to the Author

Please use the space provided to explain your answers to the questions above. You may also include additional comments for the author, including concerns about dual publication, research ethics, or publication ethics. (Please upload your review as an attachment if it exceeds 20,000 characters)

Reviewer #1: The author has addressed all the concerns raised by reviewers. I recommend acceptance toward this manuscript. The author has done a nice job and has made importation contributions to the literature.

Reviewer #2: The paper has been significantly changed. Most comments have been addressed. The introduction informs better on the Author's contribution, the second section explains the theoretical approach, and therefore the paper's results and implications are more clear.
Nevertheless, I recommend some more corrections:
1. On p. 3 the author writes that the study aims to examine the competitiveness of informal groundwater markets. As competitiveness is not analyzed in the paper and an introduction of an additional concept may be confusing I suggest changing this sentence, eg. This study investigates the seller's and buyer's relative power to bargain while negotiating the contractual agreement in informal groundwater markets.

Answer to comment 1: Thank you for the suggestion. It is a very thoughtful suggestion, and I have incorporated it in the revise manuscript.

2. The introduction section is too long in my opinion. I suggest to delete a description of the design of the study and results (from p. 5 starting with a sentence "Individual agents’ preferences help us understand...").

Answer to comment 2: I agree it is quite long. So now I have removed the results section, completely from the introduction section.
3. Section 2 seems to be more on contracts and to start with hedonic pricing. I suggest starting with a definition of bargaining power. Section 4.2 should be incorporated into this section. It does not fit in section 4 in my opinion.

Answer to comment 3: I agree, the concept and difference between bargaining power and market power is coming bit late. Now I have moved it into section 2.

4. The last section should be titled "Discussion and conclusions"

Answer to comment 4: I have renamed section 7 into discussion and conclusion.

5. When using first-person pronouns in the article the Author sometimes writes "we" and sometimes "I" (eg. section 4.2 or. p. 30: "In this study, we examine the distributional concern in Indian informal groundwater markets. I do it by analysing..."). I suggest choosing one of them.

Answer to comment 5: Thanks, I have made it consistent now by using “I” instead of WE

6. There are some small errors so the paper needs to be checked again. Eg. p. 13: "by placing price about cost" (should be rather "above"); p. 30 "Jacoby et al., (2004) find water prices charged are found to be higher"; p.44 footnote 6, it should be "the buyer wilingness to pay" instead of "the buyer wiliness to pay".

Answer to comment 6: I

Thank you for your detailed reading. I have rectified many such discrepancies in the revised manuscript.

7. PLOS authors have the option to publish the peer review history of their article ([what does this mean?](https://journals.plos.org/plosone/s/editorial-and-peer-review-process#loc-peer-review-history)). If published, this will include your full peer review and any attached files.


If you choose “no”, your identity will remain anonymous but your review may still be made public.


**Do you want your identity to be public for this peer review?** For information about this choice, including consent withdrawal, please see our [Privacy Policy](https://www.plos.org/privacy-policy).

Reviewer #1: Yes: Jian-yu Fisher Ke

Reviewer #2: No


[NOTE: If reviewer comments were submitted as an attachment file, they will be attached to this email and accessible via the submission site. Please log into your account, locate the manuscript record, and check for the action link "View Attachments". If this link does not appear, there are no attachment files.]

While revising your submission, please upload your figure files to the Preflight Analysis and Conversion Engine (PACE) digital diagnostic tool, <https://pacev2.apexcovantage.com/>. PACE helps ensure that figures meet PLOS requirements. To use PACE, you must first register as a user. Registration is free. Then, login and navigate to the UPLOAD tab, where you will find detailed instructions on how to use the tool. If you encounter any issues or have any questions when using PACE, please email PLOS at [figures@plos.org](mailto:figures@plos.org). Please note that Supporting Information files do not need this step.

*In compliance with data protection regulations, you may request that we remove your personal registration details at any time.* [*(Remove my information/details)*](https://www.editorialmanager.com/pone/login.asp?a=r)*. Please contact the publication office if you have any questions.*
